# Appraisal of the characteristic dataset of the synthesized nanobiocomposite hydrogel

**DOI:** 10.1016/j.dib.2019.105031

**Published:** 2019-12-31

**Authors:** Hamed Tashakkorian, Vahid Hasantabar, Amrollah Mostafazadeh, Monire Golpour

**Affiliations:** a Cellular and Molecular Biology Research Center (CMBRC), Health Research Institute, Babol University of Medical Sciences, Babol, Iran; b Department of Pharmacology, School of Medicine, Babol University of Medical Sciences, Babol, Iran; c University of Mazandaran, Faculty of Chemistry, Department of Organic-Polymer Chemistry, Babolsar, Iran; d Molecular and Cell Biology Research Center, Student Research Committee, Faculty of Medicine, Mazandaran University of Medical Sciences, Sari, Iran

**Keywords:** Chitosan, Hydrogel biocomposite, MTT assay, Nano zeolite, Swelling kinetics

## Abstract

The survey on the characteristic data presented here, are related to the study entitled “Transparent chitosan based nanobiocomposite hydrogel: Synthesis, thermophysical characterization, cell adhesion and viability assay” [1]. Scanning electron microscopy images, evidence for structural confirmation and more description about biological assay are presented. The thermophysical characteristic including Differential scanning calorimetery and thermogravimetery analysis are described. Swelling kinetic parameters for the prepared hydrogel were calculated and showed that Schott's equation is well suited for explaining the swelling behavior of this transparent hydrogel.

**Table:** Specifications Table

Subject area	*Chemistry, Biology*
More specific subject area	*Transparent hydrogel preparation and characterization*
Type of data	*graph, figure*
How data was acquired	*The outcomes were provided by SEM, NMR, TGA and DSC. Also some descriptions about the water absorption kinetics as well as biological assays were presented.*
Data format	*Raw, analyzed*
Experimental factors	*FTIR of the prepared samples, DSC and TGA of the modified chitosan, also swelling kinetic were apprised.*
Experimental features	*The chitosan was functionalized and characterized using NMR and imaged by SEM technique. Then thermophysical assessments were performed using DSC and TGA protocols. Water absorption and further elongation at different temperatures were imaged.*
Data source location	*Babol university of medical sciences, Mazandaran, Iran*
Data accessibility	*Available in this article*
Related research article	*Transparent chitosan based nanobiocomposite hydrogel: Synthesis, thermophysical characterization, cell adhesion and viability assay* [1]

**Table:** 

**Value of the Data** • These data display the structural evidence for the prepared nanocomposite hydrogel, so researchers who are interested in biomedical fields can take advantage of it. • With regards to bioresearcher's interest, the reason for low cell proliferation in some mentioned cell culture medium was declared and also cell adhesion on the prepared scaffold was evaluated by gimsa staining. • The therophysical behavior of the modified chitosan as a main biopolymeric structure of this hydrogel was appraised and displayed by DSC and TGA. • The water absorption in higher temperature (37 °C) was more than room temperature as a result of more softening of the nanocomposite and for further in vivo experiments the resulted swelling kinetic parameters was calculated.

## 1 Data

**Fig. 1:**
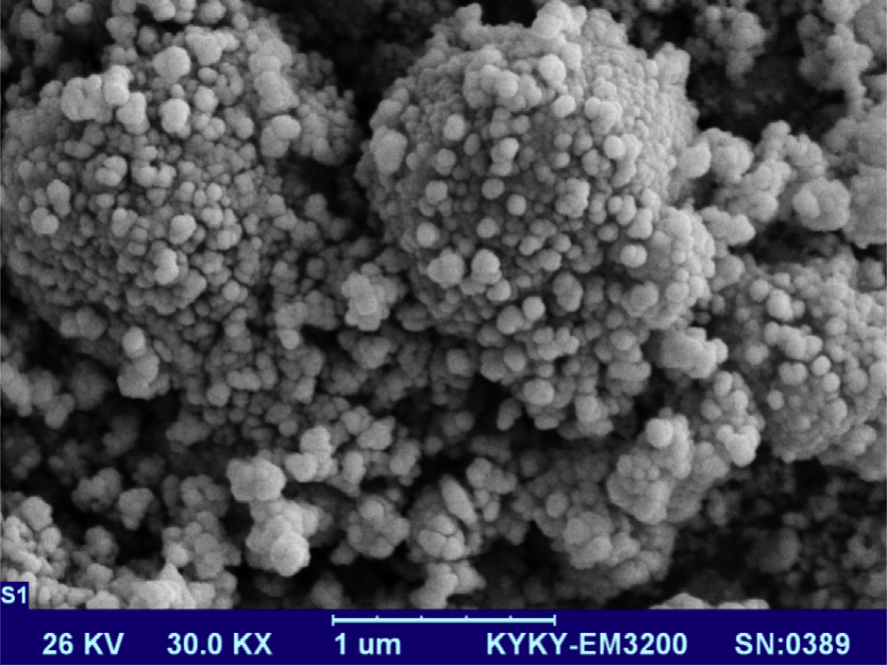
SEM images of nano sized Clinoptilolite zeolite.

**Fig. 2:**
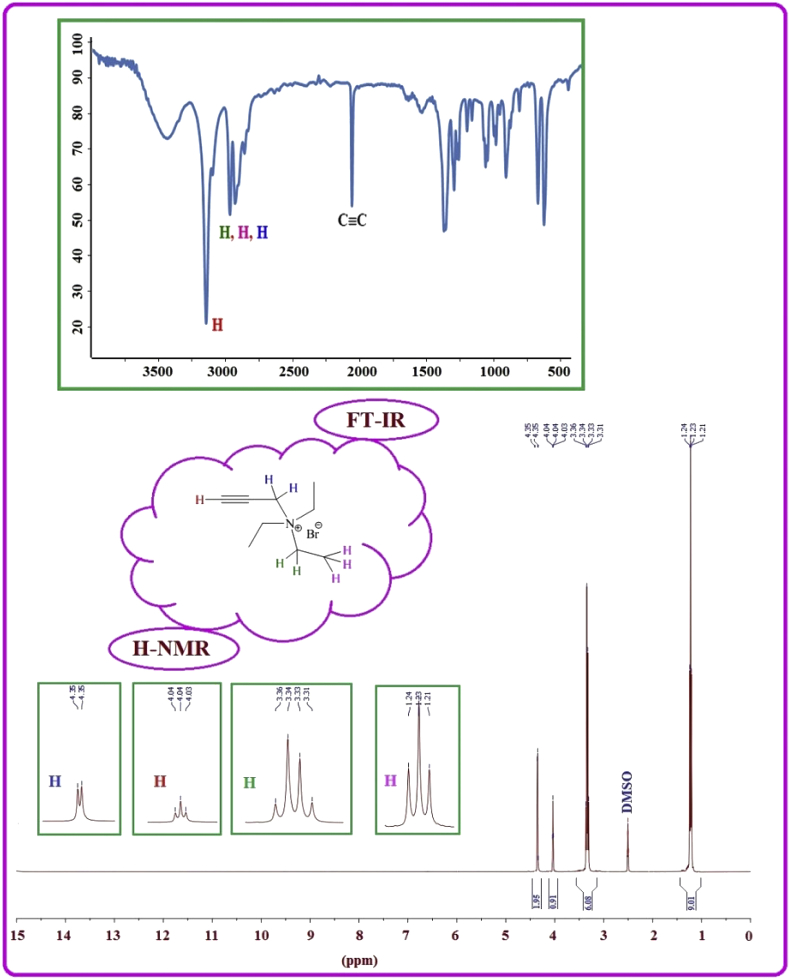
FT-IR and ^1^H NMR of propargyl triethyl ammonium bromide.

**Fig. 3:**
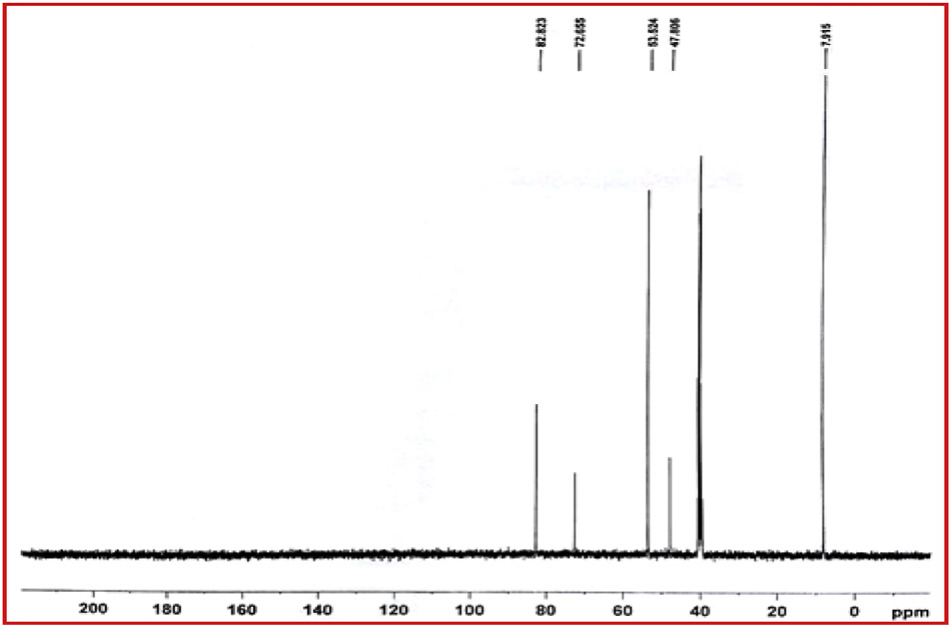
^13^C NMR of propargyl triethyl ammonium bromide.

**Fig. 4:**
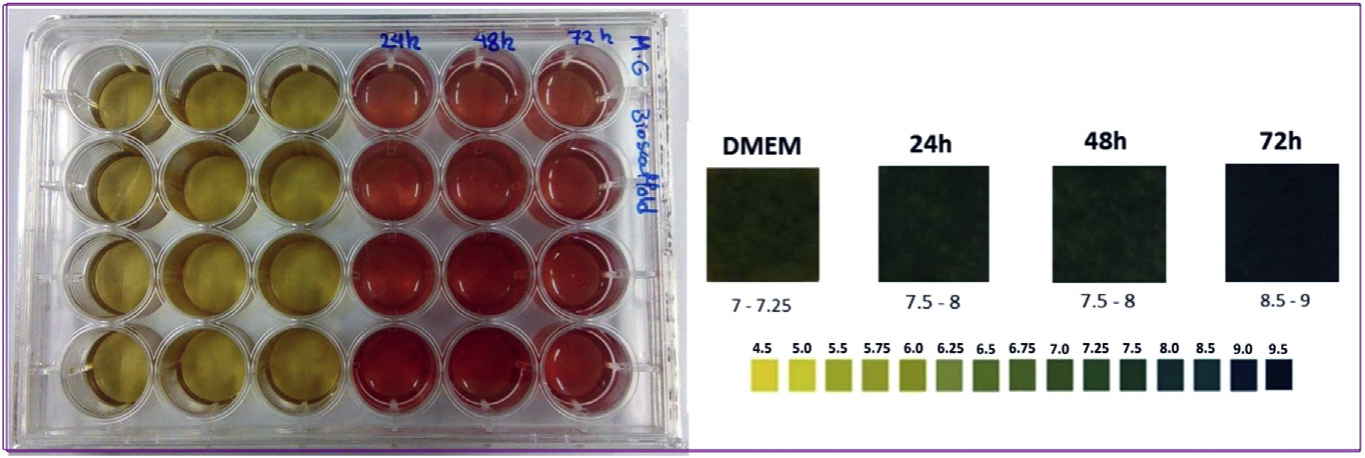
Preparation of the scaffold extracts and determination of the pH values of the 24, 48 and 72 hours extract.

**Fig. 5:**
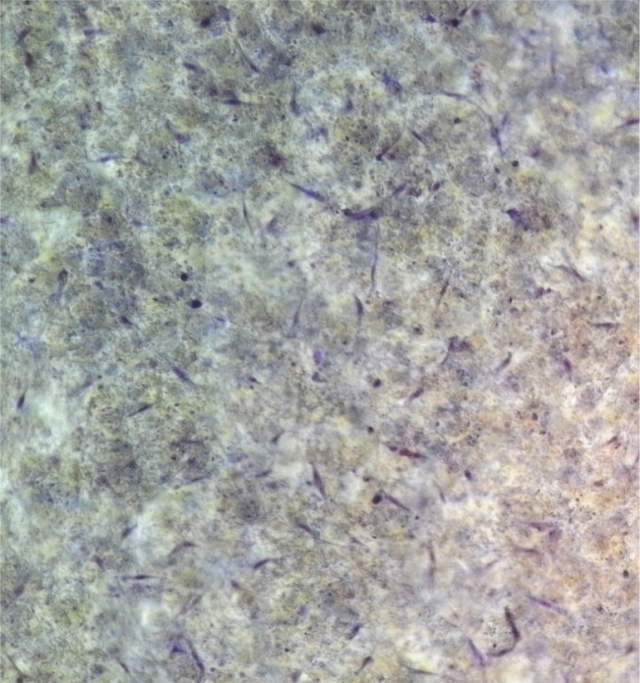
Dermal fibroblast stained with giemsa after 72 H culture under a light microscope.

**Fig. 6:**
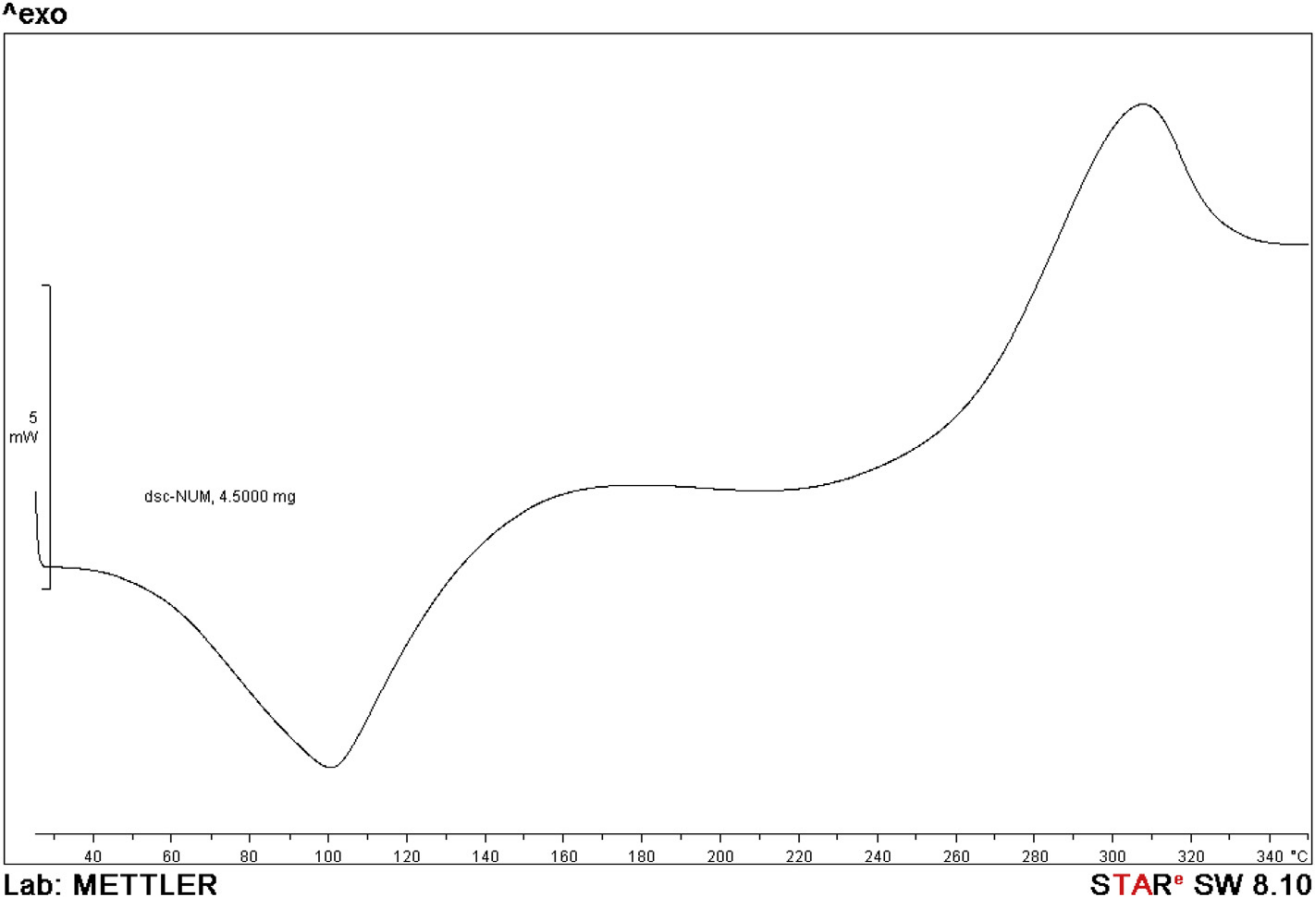
DSC curve of modified chitosan.

**Fig. 7:**
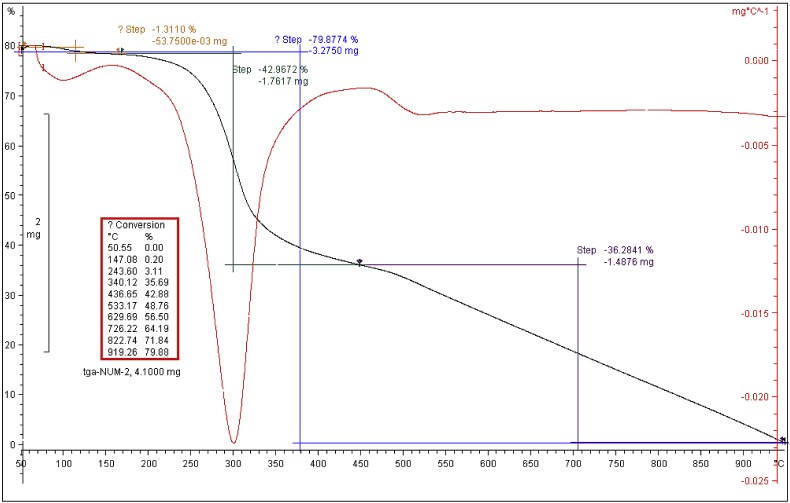
TGA curve of modified chitosan.

**Fig. 8:**
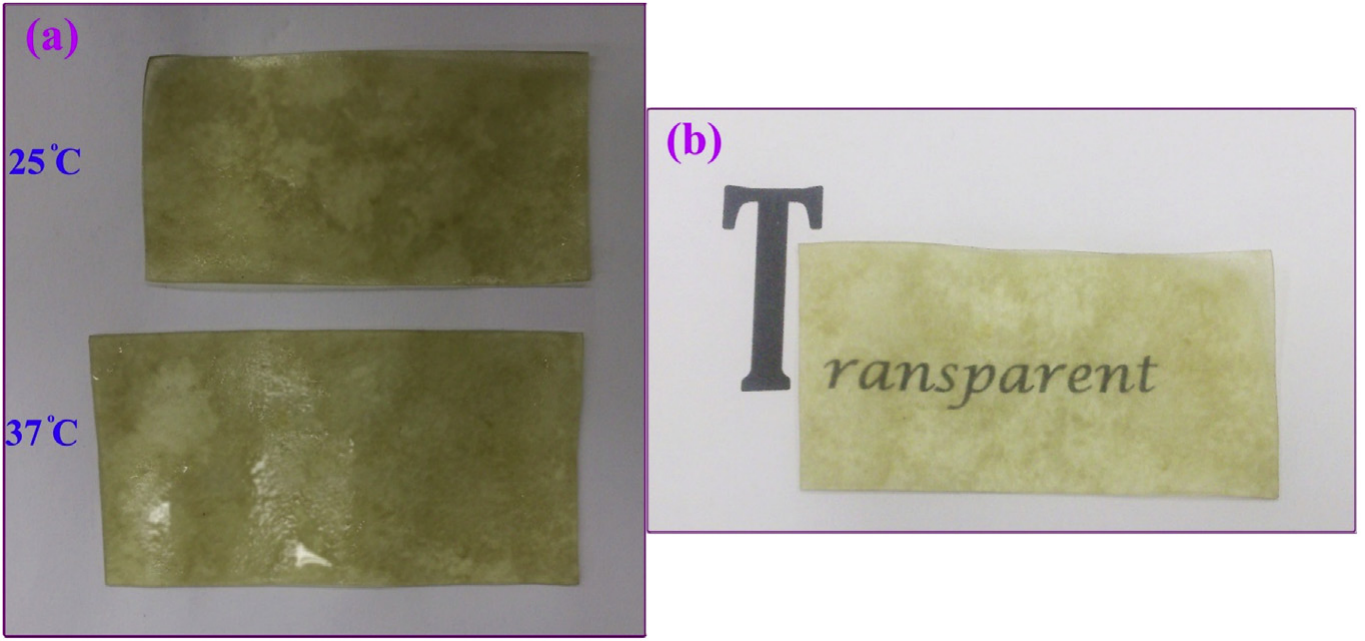
(a) difference in dimension of the same sized hydrogel after swelling for 24 hours. (b) Transparency of the hydrogel.

**Fig. 9:**
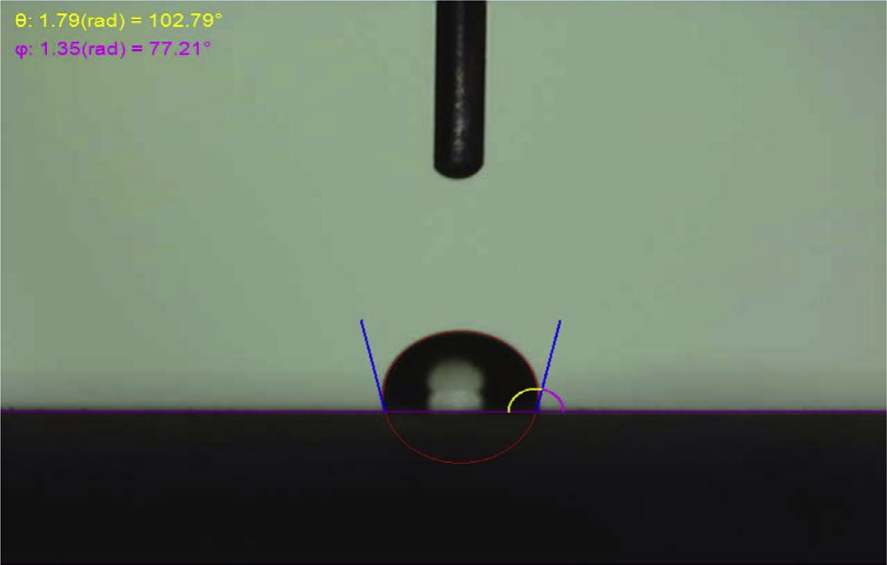
The static water contact angle (WCA) of the prepared hydrogel.

NMR spectrum including ^1^HNMR and ^13^CNMR spectrum of functionalized chitosan were recorded on a Bruker 400 MHz. Fig. 1 displays the previously reported clinoptilolite nanoparticle [2] which was imaged by SEM. FT-IR and ^1^H NMR of propargyl triethyl ammonium bromide were presented in Fig. 2. Also, ^13^CNMR which is so important in order to find the carbon structure of the compound was displayed as Fig. 3. To better understanding the chemical structure, chemical shifts of the ^1^H NMR and ^13^C NMR as well as splitting of the mentioned peaks were inserted in Table 1. Preparation of the scaffold extracts and determination of the pH values of the 24, 48 and 72 hours extract and also gimsa staining was demonstrated in Fig. 4, Fig. 5 respectively. Moreover thermophysical analyses (Fig. 6, Fig. 7) were done on a Perkin-Elmer Pyris Diamond and Pyris 6 TGA Consumables. Difference in dimension of the same sized hydrogel in two temperatures and the transparency of the hydrogel were displayed in Fig. 8. The static water contact angle (WCA) was determined by Sessile drop measurements using a Contact Shape Analyzer, CA-500M (Sharifsolar, IRAN) (Fig. 9). Swelling kinetic parameters for the prepared chitosan based hydrogel were presented in Table 2.

**Table 1:** ^1^H NMR and ^13^C NMR dataset of propargyl triethyl ammonium bromide.

	Chemical shift (ppm)/splitting				
^1^H-NMR	1.23/triplet	3.33/quartet	4.04/triplet	4.35/doublet	**–**
^13^C-NMR	7.91	47.80	53.52	72.45	82.82

**Table 2:** Second-order swelling kinetic parameters for the prepared chitosan based hydrogel.

Hydrogel	${\text{S}}_{\text{eq}}^{\text{Calc.}}$	K_2_ × 10^5^ (min^−1^)	R^2^
25 °C	156.25	42.845	0.999
37 °C	222.22	89.603	0.999

## 2 Experimental design, materials and methods

The clinoptilolite (CP) nanoparticles which were incorporated in this nanobiocomposite were prepared according to the previous published article [2]. The spherical structure and the size of nanoparticles were shown with SEM image in Fig. 1. To prepare modified chitosan with more applicable characteristics, functionalization of chitosan was done with triethyl amine and propargyl bromide. Through this process, quaternary ammonium moiety was inserted and the hydrophilicity of the chitosan was improved. The FTIR, ^1^HNMR (Fig. 2) and ^13^CNMR (Fig. 3) spectrum depicted the clear evidence of the modification. The characteristic peaks were demonstrated in details.

As can be seen in the Fig. 4, the pH values of the scaffold extracts were increased after 48 hours. This elevation from 7.5 up to 9 was not suitable for cell culture and was resulted in low cell proliferation.

To demonstrate the cell adhesion and proliferation on the scaffold surface after mentioned time, gimsa staining as well as MTT assay was performed. The purple fibroblast cells are evident in the microscopic image of the scaffold surface (Fig. 5).

Thermal properties of modified chitosan were evaluated by DSC and TGA experiments. DSC of the prepared chitosan exhibited a broad exothermic peak at 315 °C (Fig. 6). Moreover, TGA experiments of the modified chitosan displayed that the start of degradation was occurred at around 230 °C and ended at 340 °C (Fig. 7). During this thermal process the sample lost about 42.97% of its weight. This step was mainly due to the decomposition and degradation of the functionalized moieties such as etheric and amine bonds and also polymeric chain [3]. According to the thermogram, the maximum weight loss is about 305 °C. This degradation and elimination step in TGA was displayed by exothermic peak in DSC thermogram too. The DSC is showing an endothermic peak at about 100 °C that may be correlated to removal of loosely bound water and existing solvent in the polymeric network.

Water absorption properties were assayed in two temperatures; in normal body temperature (37 °C) and room temperature (25 °C). The clear difference in size was occurred (Fig. 8a). The data obtained from this evaluation were presented in “Transparent chitosan based nanobiocomposite hydrogel: Synthesis, thermophysical characterization, cell adhesion and viability assay” [1] article. Also the transparency of the hydrogel was displayed in Fig. 8b. The static water contact angle (WCA) for the prepared chitosan based hydrogel was determined by (Krüss, Hamburg, Germany) (Fig. 9).

To survey on further experiments, some critical swelling kinetic parameters were calculated [4,5] and showed that Schott's equation can explain this swelling behavior of the modified chitosan hydrogel (Table 2). The values obtained for *K*_*2*_, illustrated the effect of temperatures on water absorption properties of hydrogels.
